# Drugs with glutamate-based mechanisms of action in psychiatry

**DOI:** 10.1007/s43440-024-00656-8

**Published:** 2024-09-27

**Authors:** Adrian Andrzej Chrobak, Marcin Siwek

**Affiliations:** 1 Department of Adult Psychiatry, Jagiellonian University Medical College, Kopernika 21A, 31-501 Kraków, Poland; 2 Department of Affective Disorders, Jagiellonian University Medical College, Kopernika 21A, 31-501 Kraków, Poland

**Keywords:** Excitotoxicity, Psychosis, Mood disorders, Treatment resistance, Antidepressant drugs, Antipsychotic drugs

## Abstract

Psychopharmacotherapy of major psychiatric disorders is mostly based on drugs that modulate serotonergic, dopaminergic, or noradrenergic neurotransmission, either by inhibiting their reuptake or by acting as agonists or antagonists on specific monoamine receptors. The effectiveness of this approach is limited by a significant delay in the therapeutic mechanism and self-perpetuating growth of treatment resistance with a consecutive number of ineffective trials. A growing number of studies suggest that drugs targeting glutamate receptors offer an opportunity for rapid therapeutic effect that may overcome the limitations of monoaminergic drugs. In this article, we present a review of glutamate-modulating drugs, their mechanism of action, as well as preclinical and clinical studies of their efficacy in treating mental disorders. Observations of the rapid, robust, and long-lasting effects of ketamine and ketamine encourages further research on drugs targeting glutamatergic transmission. A growing number of studies support the use of memantine and minocycline in major depressive disorder and schizophrenia. Amantadine, zinc, and *Crocus sativus* extracts yield the potential to ameliorate depressive symptoms in patients with affective disorders. Drugs with mechanisms of action based on glutamate constitute a promising pharmacological group in the treatment of mental disorders that do not respond to standard methods of therapy. However, further research is needed on their efficacy, safety, dosage, interactions, and side effects, to determine their optimal clinical use.

## Introduction

Psychopharmacotherapy of affective and other mental disorders is mostly based on drugs that modulate serotonergic, dopaminergic, or noradrenergic neurotransmission, either by inhibiting reuptake or by acting as agonists or antagonists on specific monoamine receptors [1]. Treatment based on the abovementioned mechanisms has many limitations. Firstly, it is associated with a significant delay of the therapeutic effect, and secondly, it is not effective in the case of drug resistance, as the application of subsequent pharmacological strategies, based on the same mechanisms, results in decreasing efficacy with each therapy cycle and may exacerbate the drug resistance by inducing adaptive changes in the synapses [2–4]. A solution to the abovementioned problems is the development of drugs that target glutamatergic neurotransmission [5]. Especially in the case of depressive disorders, drugs targeting glutamate receptors (such as ketamine, which is a prototypical example) offer an opportunity for a rapid therapeutic effect and overcoming drug resistance, with minimal risk of adverse effects generated by conventional drugs [5]. Modulation of glutamatergic receptor activity is also used to augment classical antidepressant treatment (e.g. by adding esketamine, zinc, minocycline, or amantadine) [5]. Some plant-derived compounds - e.g. safranal also affect the activity of glutamatergic receptors, which results in antidepressant or anxiolytic effects [6, 7]. Apart from affective disorders, it has been shown that drugs targeting glutamate receptors, such as memantine, can improve the outcomes of treatment of obsessive-compulsive disorders (OCD) or schizophrenia (SZ), enhancing the action of other drugs [8–12]. Additionally, some mood stabilizers, such as lamotrigine or valproic acid, alongside many other complex mechanisms, exert their action by modifying glutamatergic neurotransmission. In this article, we aim to conduct a PubMed review of selected glutamate-modulating drugs that are already used in psychiatric practice or are at the stage of advanced clinical trials. We will discuss their mechanism of action, as well as preclinical and clinical studies of their efficacy in treating mental disorders.

## Glutamate receptors

Glutamate is the predominant excitatory neurotransmitter in the human central nervous system, which plays a vital role in synaptic plasticity, including long-term potentiation [13, 14]. Glutamate receptors are classified into two types: ionotropic glutamate receptors (iGluRs) and metabotropic glutamate receptors (mGluRs). IGluRs consist of N-methyl-D-aspartate receptor (NMDAR), α-amino-3-hydroxy-5-methyl- 4-isoxazole propionic acid (AMPAR), and kainite receptors. mGluRs are divided into three groups (I, II, and III) [13]. NMDAR is a tetrameric ion channel that consists of two essential GluN1 subunits along with two others, which can either be GluN2 or GluN3 [15]. Activation of this receptor requires bindings of either two glycine or d-serine molecules (acting as co-agonists) to its GluN1 subunits, plus two glutamate molecules to its GluN2 subunit [15]. NMDAR isis functionally paired with AMPAR [16]. Activation of the latter causes celldepolarization, which results in the displacement of the Mg^2+^ ion from the NMDAR pore and the subsequent entry of Ca^2+^ and Na^+^ ions into the neurons [16]. An elevated intracellular Ca^2+^ concentration is essential for the proper functioning and structural integrity of neurons [17]. However, in pathological circumstances, excessive influxes of Ca^2+^ in neuronal deterioration or death [17]. This phenomenon, referred to as excitotoxicity is a key pathophysiological mechanism of neurodegeneration in major psychiatric disorders [18]. There is an increasing amount of research suggesting that altered glutamatergic neurotransmission emerges as a vital pathophysiological event related to etiopathogenesis of mental disorders such as SZ, OCD, bipolar disorder (BD), and major depressive disorder (MDD), encompassing instances of treatment-resistant depression (TRD) [18].

### Ketamine and (S)-ketamine

Ketamine is a drug with a long history in anesthetic practice spanning approximately six decades that has demonstrated a notably promising profile as an antidepressant over the last twenty years [19]. The main mechanism of action of this drug relies on non-competitive inhibition of NMDAR, coupled with enhanced stimulation of AMDAR [5]. It is hypothesized that antidepressant effect of this substance is related to the cascade of events related to glutamatergic disinhibition [5]. Through rapid blockade of NMDAR, ketamine inhibits GABAergic interneurons responsible for tonic inhibition of cortical glutamatergic neurons [5]. Increased release of glutamate by these cells activates AMPAR, leading to an influx of Ca^2+^ and initiation of Ca^2+^-dependent processes, which result in increased synthesis and release of brain-derived neurotrophic factor (BDNF). Finally, BDNF induces intracellular pathways mediated by the mammalian target of rapamycin (mTOR) and extracellular regulated kinase (ERK). Those processes lead to increased synthesis of synaptic proteins resulting in long-lasting neurostructural and neurofunctional changes [5].

A meta-analysis has shown that in TRD, even a single dose of ketamine can induce a rapid and robust antidepressant effect lasting up to seven days [20]. The network meta-analysis of 31 randomized controlled trials has shown that two weeks after the initiation of the treatment, ketamine was ranked first among twelve pharmacological and two somatic interventions for TRD, reaching a 14-fold response rate versus placebo [20]. Noteworthy, within 40–240 min after the first administration, ketamine induces a significant reduction of suicidal ideation that lasts up to one week. Repeated administration of this drug has been shown to completely alleviate suicidal ideation in 69% of TRD patients [21].

Although most patients experience symptom improvement for several weeks following a single ketamine infusion, a substantial number of patients present recurrence of depressive symptoms [22]. About one-fourth of those who achieved remission 72 h after the first dose, relapses within seven days post-infusion, and another fourth after the end of the second week [22]. Therefore, despite the significant effectiveness of this drug, the necessity of its administration through repeated intravenous infusions limits the possibility of its use in long-term therapy. Studies have shown that the S (+) enantiomer of ketamine (esketamine), which can be applied in the form of nasal spray, demonstrates similar efficacy and safety pattern in the treatment of TRD patients [23]. A randomized double-blind active-controlled study has shown that esketamine administered twice weekly presented a clinically meaningful effect during the second day of treatment that persisted after four weeks of trial [24]. Improvement of depressive symptoms has been shown to persist 2 months after the cessation of esketamine dosing [25]. A growing number of studies demonstrates that esketamine is safe and effective add-on to antidepressant treatment of TRD resulting in significant increase of remission rates in this clinical group [26]. The discovery that NMDAR antagonists may induce rapid onset and long-lasting antidepressants effects is a breakthrough in the modern psychopharmacotherapy, that has led to increased interest in the use of glutamatergic compounds in the treatment of major psychiatric disorders [5, 18].

### Memantine

Memantine is a low affinity, noncompetitive NMDAR antagonist that can easily cross the blood-brain barrier that is primarily used in treatment of dementia [27]. Preclinical studies demonstrated that this drug poses antidepressants activity attributed to BDNF upregulation, mitochondrial protection and anti-neuroinflammatory properties [28, 29]. A recent comprehensive systematic review and meta-analysis of 11 randomized controlled trials involving MDD, BD and SZ patients showed that memantine, primarily as an add-on treatment, notably outperformed placebo in reducing severity of depressive symptoms in mood disorders [12].

Growing number of clinical studies indicate that memantine appears to be safe and well tolerated treatment option for SZ symptoms [8]. Considering hypothesis of NMDAR hypofunction in SZ, it is controversial why memantine treatment does not yield severe side effects like other NMDAR antagonists such as ketamine or phencyclidine (PCP) [22]. It is hypothesized that memantine inhibits NMDAR based on combination of fast unblocking kinetics and strong functional voltage dependency [8]. Under physiological conditions, NMDARs are activated only by high concentration of glutamate that coincide with strong neuronal depolarisation, which is required to remove Mg^2+^ from their channel pores [8]. However, under pathological conditions, even a moderate depolarization can dislodge Mg^2+^ and allow longer stimulation, by lower glutamate concentrations [8]. While PCP reveals a high affinity to NMDAR in both physiological and pathological conditions, memantine binds weakly to these receptors in a voltage-dependent manner [8]. Memantine inhibits NMDAR only in a state of moderate depolarization, thus it prevents its prolonged activation under pathological conditions [8]. During strong depolarisation memantine quickly unblocks NMDAR channel pores, which suggest its minimal impact on physiological glutamatergic transmission and corresponds with the good tolerability and safety profile of this drug (for detailed review [8]). Meta-analysis of eight randomized, double-blind, placebo-controlled trials showed that adjunctive memantine is an effective and safe intervention for improving cognitive deficits and negative symptoms in SZ [30]. Hassanpour et al. 2019 have shown that eight weeks of memantine add-on treatment improved verbal memory, working memory, learning, phonological fluency, and learning without the effect on psychotic symptoms in the chronic SZ patients’ group [10]. Veerman et al. 2016 performed a randomized, double-blind placebo-controlled study of memantine augmentation in clozapine-refractory SZ [31]. When compared to the placebo group, twelve weeks of memantine treatment was associated with improved verbal and visual memory and negative symptoms [31]. The authors hypothesized that the synergistic effects of those drugs may stem from their collective impact on NMDAR [31]. While clozapine increases the expression of both AMPAR and NMDAR [32, 33], memantine can promote additional up-regulation of NMDAR, which results in stronger neuronal activation when presented with robust stimuli [34]. This mechanism may facilitate the induction of long-term potentiation and improve cognitive functioning [31]. Given the fact that 30–50% of SZ patients do not respond to at least two subsequential antipsychotic drugs and up to 70% of patients treated with clozapine reach only partial response, there is a vital need to seek new strategies to overcome treatment resistance in this clinical group [35, 36]. The abovementioned studies demonstrate that memantine yields the potential to improve cognitive and negative symptoms in treatment-resistant SZ.

A growing number of studies indicate that excessive glutamatergic activity, elevated glutamate levels in cerebrospinal fluid, and gene polymorphisms coding for the NMDAR may play a significant role in the pathophysiology of OCD [9, 11, 37–39]. A meta-analysis of eight clinical studies has shown that eight weeks of memantine augmentation to first-line pharmacological treatment resulted in a significant reduction of OCD symptoms (mean reduction of 11.73 points on the Yale-Brown Obsessive-Compulsive Scale) [11]. Further analyses of four double-blind placebo-controlled trials revealed that patients receiving memantine had up to a 3.61-fold higher response rate than the placebo group [11]. Memantine augmentation has been proposed as an intervention for selective serotonin reuptake inhibitors-resistant OCD or BD patients who exhibit contradiction to the use of first-line treatment [11].

### Minocycline

Minocycline is a tetracycline antibiotic that effectively penetrates the blood-brain barrier and demonstrates significant neuroprotective properties [40]. This drug has been shown to inhibit nitric oxide synthesis, suppress proinflammatory activity of microglia, and reduce apoptosis [41–44]. A growing number of studies indicate this drug may directly affect glutamatergic transmission [43, 45, 46]. Recently it has been proposed that minocycline positively modulates GluR1 subunit receptors of AMPA leading to the BDNF upregulation [46, 47]. While this drug does not present direct interactions with NMDAR it has been shown that minocycline can attenuate glutamate-induced Ca^2+^ influx and prevent neuronal death caused by excitotoxicity [46]. Clinical and animal studies suggest that the abovementioned mechanisms may be related to the broad effects of this drug in the treatment of SZ and MDD [46]. Minocycline has outperformed placebo in the reduction of depressive symptoms in the groups HIV positive patients and TRD group, as well as in the improvement of global functioning scores in MDD [48–50]. However, recent studies did not replicate those findings [49]. Hellmann-Regen et al. (2022) haveperformed a multicentre double-blind randomized clinical trial of 200 mg minocycline as an add-on to antidepressant treatment in MDD of throughout six weeks with a six-month follow-up [51]. In the group of 173 participants, the drug did not alter the severity depressive of depressive symptoms [51]. The discrepancy between the abovementioned studies may be partially explained by the observation that minocycline efficacy is mediated by the baseline inflammatory state [52]. In a double-blinddouble-blind randomized clinical trial, it has been shown that minocycline responders presented increased serum concentration of Il-6 and low-grade inflammation defined as CRP ≥ 3 mg/l [52]. Thus, the antidepressant effect of minocycline may be restricted to depression which is associated with increased inflammation [52].

Clinical studies evaluating the efficacy of minocycline add-on treatment to both typical and atypical antipsychotics reveal improvement in the wide spectrum of SZ symptoms [46, 53, 54]. In a double-blind, randomized placebo-controlled study, Levkovitz et al. (2010) haveshowed that minocycline augmentation of antipsychotic treatment in the group of early SZ patients resulted in significant improvement of negative symptoms and executive functions [54]. In an open-label study, minocycline add-on to atypical antipsychotic drugs resulted in approximately 40% reduction of both positive and negative symptoms, and a 50% decrease in general psychopathology measured by PANS after four weeks of the treatment [53]. Those results suggest that minocycline yields the potential to ameliorate positive, negative, and cognitive symptoms in SZ.

## Zinc

Zinc serves as an essential cofactor of many proteins and plays a crucial role in the processes of DNA replication, transcription, protein synthesis, maintaining cell membrane integrity, and immune system regulation [55]. This ion is a vital component of the antioxidative mechanisms responsible for the stabilization of the blood-brain barrier and neuronal survival [56, 57]. Zinc has been shown to play a significant role in the pathophysiology of depression, related to its modulatory effect on glutamatergic transmission and synaptic plasticity [58]. Zinc is a potent regulator and inhibitor of the NMDAR, an antagonist of mGluR1 and 2 receptors, and an enhancer of AMPAR activity [59]. Its deficiency leads to overactivity and upregulation of NMDAR posing a threat of excitotoxicity [59] and it is related to the occurrence of depressive symptoms [57].

Antidepressant properties of zinc have been shown in both clinical and animal studies. This trace element was found to be active in various models of depressive-like behavior, such as the tail suspension test, the forced swim test, and chronic unpredictable stress, olfactory bulbectomy, and chronic mild stress [60–62]. Moreover, zinc administration has been shown to enhance the antidepressant effect of citalopram and imipramine [56, 60, 61]. In our placebo-controlled study zinc supplementation potentiated imipramine treatment leading to a significant reduction of depressive symptoms in TRD, but not in the group of non-resistant MDD patients [63]. Another placebo-controlled, double-blind trial demonstrated that after six and twelve weeks of zinc add-on treatment to tricyclic antidepressants or selective serotonin reuptake inhibitors led to a significant reduction of depressive symptoms measured with the use of the Hamilton Depression Rating Scale and Beck Depression Inventory [63]. This effect has been replicated in further studies in MDD [64, 65].

## Crocus sativus

Saffron (*Crocus sativus*) is a commonly used herb for coloring and flavoring food. It has been suggested that this herbal supplement possesses an antidepressant effect associated with its impact on the monoaminergic system [6, 66, 67]. Two of its major bioactive compounds, which are crocin and safranal may inhibit reuptake of serotonin, dopamine, and norepinephrine [66]. Saffron ' effects may be also mediated by their interactions with the glutamatergic system. Acute administration of safranal reduced kainic acid-induced elevation of glutamate transmission in rat hippocampus [68]. Moreover, Saffron extracts decreased glutamatergic signalization in rat cortical slices [69] which suggests that this substance may ameliorate the excessive release of this neurotransmitter. A preclinical study has shown that safranal, crocins, and crocetin may have depressive-like symptoms in mice measured in forced swim tests [70, 71]. Meta-analysis of five randomized clinical trials has shown that saffron supplementation was superior to placebo in the reduction of depressive symptoms [72]. Short-term six-week studies have demonstrated that there are no significant differences between saffron and imipramine or fluoxetine in MDD patients [72–74]. However, it should be noted that all of the abovementioned studies were single-centre trials organizedorganized within the same clinical settings in Iran, which indicates a need for more multisite studies [72]. Further randomized placebo-controlled clinical trials have shown that saffron as well as mixed saffron/curcumin preparations are superior to placebo in ameliorating depressive symptoms in MDD [67, 75]. In a group of older MDD patients, saffron has revealed an antidepressant effect comparable to sertraline [76]. More large-sample, multisite, and long-term clinical trials are needed to clarify saffron’s potential role in MDD treatment [72]. Despite the methodological limitations of these studies, the Canadian Network for Mood and Anxiety Treatments (CANMAT) enlisted saffron as a third-line option of so-called “complementary and alternative medicine treatments” [77]. Based on the observations that saffron extracts may limit excessive glutamatergic transmission, it has been suggested that this herbal medicine may be effective in the treatment of SZ [78]. Animal studies have reported that acute supplementation with crocins improved the schizophrenia-like negative symptoms induced by sub-chronic treatment with ketamine [78]. Interestingly, this intervention has also decreased the severity of ataxia symptoms, which are observed in SZ patients [79, 80]. Currently, there is only one double-blind placebo-controlled study that analyzed the effects of saffron extracts and crocin in SZ [81]. However, this trial evaluated only the safety and tolerability of those substances but did not include any efficacy measures [81]. Therefore, there is no data indicating the validity of using saffron for the treatment of SZ symptoms [81].

### Amantadine

Amantadine is a drug primarily known for its use in the treatment of type A influenza virus and dyskinesia associated with parkinsonism, but a growing number of studies indicate the potential use of this drug in the therapy of mental disorders, especially in affective disorders [82–85]. Amantadine may reduce the severity of depressive symptoms by increasing dopaminergic transmission through the inhibition of dopamine transporters (DAT), monoamine oxidase B (MAO-B), and agonistic effect on D_1_ [83, 86, 87]. Moreover, this drug may potentially exert antidepressant effects through noncompetitive inhibition of NMDAR, which can both enhance the inhibition of DAT and induce effects like those of esketamine and ketamine [83, 86, 87]. Rogóz et al. 2007 have shown that amantadine exerts a significant antidepressant effect which may be used in the treatment of TRD [84]. Amantadine augmentation to ongoing imipramine therapy resulted in significant improvement of depressive symptoms (68% of responses and partial responses) after six months of joint treatment [84]. Krzystanek et al. 2023 presented the possibility of using this drug in the treatment of dysthymia [84]. In a naturalistic case series study, amantadine has been shown to be an effective and well-tolerated drug associated with quick improvement of depressive symptoms and persistency of the therapeutic effect after the discontinuation of the treatment [83]. The effectiveness of this therapy was comparable to sertraline [83]. Another study of this group has demonstrated the utility of amantadine as an add-on therapy for treatment-resistant bipolar depression [85]. In the series of four patients, it has been shown that the use of this drug resulted in rapid improvement of depressive symptoms after one week of treatment without the switch of the phase [85]. This effect persisted even when the drug was discontinued two weeks after the antidepressant effect was achieved [85]. In the retrospective chart review, Morrow et al. 2021 evaluated the utility of amantadine for the treatment of childhood and adolescent psychiatric symptoms in the group of 297 pediatric patients with a variety of clinical diagnoses (attention deficit hyperactivity disorder, conduct disorder, depressive disorder, anxiety disorder, autism spectrum disorder, post-traumatic stress disorder, intellectual disability) [88]. Amantadine treatment was effective in improving impulsivity, irritability, anger, and concentration [88]. Therapy with this drug enabled discontinuation of antipsychotic or antidepressant drugs in 42% and 28% of patients [89], respectively [88] Abovementioned results indicate the need for randomized controlled clinical trials evaluating the utility of the amantadine in the treatment of mental disorders.

### Antiseizure medications

Due to their significant mood-stabilizing effect, antiseizure medications have a wide range of applications in the treatment of mental disorders [90]. Those drugs are commonly used as normothermic medication for acute manic states (carbamazepine and valproate), acute bipolar depression (lamotrigine), and in long-term prevention of affective episodes (carbamazepine, valproate, and lamotrigine) in BD [89, 91, 92]. In terms of MDD, a meta-analysis of eight double-blinded randomized controlled trials has shown that lamotrigine augmentation of antidepressantantidepressant treatment results in a significant reduction of depressive symptoms in TRD [93]. Topiramate is an antiseizure drug with a complex mechanism involving AMPAR and kainite receptor inhibition [94]. A recent meta-analysis confirmed that topiramate significantly reduces both binge eating frequency and weight in patients with binge eating disorder [94]. In the case of SZ, adjunctive use of this drug to antipsychotic medication has been shown to significantly improve positive, negative, and general symptoms as well as resulted in weight reduction [95]. Each of the above-mentioned drugs significantly affects the glutamatergic system. The description of these mechanisms is presented in Table 1.

**Table 1:** Mechanisms of action of drugs targeting glutamatergic transmission used in the treatment of major psychiatric disorders [3, 7, 41, 43, 58, 110, 111]

Substance /drug	Mechanisms of action related to glutamate	Other mechanisms of action	Most important adverse effects /clinical limitations	References
Valproic acid	• ↓ excitatory glutamatergic transmission and ↑ inhibitory GABAergic transmission associated with the following mechanisms: • positive allosteric modulation GABA-A receptor • GABA transaminase inhibition → ↓ GABA degradation • ↑ activity of glutamate acid activity→ ↑ glutamate degradation and ↑ glutamate to GABA conversion • inhibition of Na + channels and voltage-dependent Ca^2+^ channels, resulting in inhibition of glutamate release	• inhibition of histone deacetylases • non-competitive inhibition of microsomal acyl-CoA synthetase • inhibition of phosphatidylinositol cycle, e.g., by reducing MIPS activity • modulation of Ca^2+^ channel activity of type T, type L, and type N, • ↓ activity of COX-1 and COX-2 within CNS • inhibition of mitochondrial dehydrogenases • regulation of cyclin-dependent kinase inhibitor 1 • downregulation of programmed death receptor ligand 1 (PD-L1)	The most common side effects are increased appetite and body weight, dizziness, ataxia, drowsiness, fatigue, gastrointestinal symptoms, rash, tremors, hair loss Beware of the risk of: • hepatotoxicity • induction or worsening of metabolic syndrome symptoms • hematological disorders (including thrombocytopenia) • pancreatitis Administered to pregnant women may cause serious birth defects and an increased risk of neurodevelopmental disorders in children exposed to the drug in utero	[3, 113− 117]
Lamotrigine	• inhibition of Na + channels and voltage-dependent Ca^2+^ channels, resulting in inhibition of glutamate release • weak inhibition of AMPA1R	• ↓ neuronal excitability and firing frequency due to inhibition of Na^+^ and Ca^2+^ channels. • modulation of HCN channels (cyclic nucleotide-gated channels) • inhibition of pro-inflammatory microglial activity • interaction with the activity of numerous receptors in the CNS (the significance of these mechanisms is unclear) - including adenosine, 5HT_3_, 5HT_2a_, GABA-A, HA, kappa and many others	Generally well-tolerated The most common side effects observed in psychiatric studies: gastrointestinal symptoms, rash, dizziness, headache, memory or concentration problems, sedation Beware of the risk of Stevens-Johnson syndrome	[3, 118–125]
Carbamazepine	• inhibition of Na + channels and voltage-dependent Ca^2+^ channels, resulting in inhibition of glutamate release	• ↓ neuronal excitability and firing frequency due to inhibition of Na^+^ and Ca^2+^ channels. • ↓ activity and production of AA-selective cPLA2 • ↓ activity of cyclooxygenases in CNS	The most common side effects are: dizziness, ataxia, drowsiness, fatigue, gastrointestinal symptoms, rash Beware of the risk of • Hyponatremia • Neutropenia/agranulocytosis • Stevens-Johnson syndrome • Hepatotoxicity • Risk of loss or reduced effectiveness of many drugs due to enzymatic induction (carbamazepine is an inductor of P-glycoprotein, CYP450 1A2, 2D6, 3A4, 2C9, 2C19) Administered to pregnant women may cause serious birth defects	[121, 126, 127]
Topiramate	• inhibition of Na + channels and voltage-dependent Ca^2+^ channels, resulting in inhibition of glutamate release • antagonism against glutamatergic kainate receptors	• agonism against alpha1 subunit of GABA-A receptors	Paresthesia, taste perversion, upper respiratory tract infection, memory and concentration difficulties occurred significantly more frequently among the topiramate-treated participants than the placebo-treated subjects in studies of binge-eating disorder patients In schizophrenia patients studies Topiramate was associated with significantly more adverse drug reactions than the control condition, including concentration/attention difficulties, psychomotor slowing, and paresthesia An increased incidence of mood disorders and depression, cases of a syndrome consisting of sudden myopia and secondary angle-closure glaucoma and cases of hyperchloremic metabolic acidosis has been reported in patients taking topiramate Administered to pregnant women may cause serious birth defects and an increased risk of neurodevelopmental disorders in children exposed to the drug in utero	[3, 128–134]
Ketamine /esketamine	• noncompetitive NMDAR antagonism	• potentiation of 5HT3_A_ receptors • antagonism of alpha-7 subunit of neuronal acetylcholine receptor • partial agonism of D_2_ receptors • cholinesterase inhibitor • inhibitor of brain nitric oxide synthase • inhibition of norepinephrine transporter	Ketamine or esketamine intravenous administration is associated with short-lasting, reversible side effects during the 4 h after the infusion: drowsiness, dizziness, poor coordination, blurred vision, dissociative or depersonalization symptoms, anxiety, transient increases in pulse and mean blood pressure, nausea, and sedation. Studies involving nasal administration of esketamine have shown the following short-term and self-limiting side effects: nausea, dizziness, vomiting, vertigo, dysgeusia, lethargy, dysesthesia, increased blood pressure, sedation, dissociation symptoms. The drugs should not be used by patients for whom increased blood pressure or intracranial pressure pose a serious risk. There is a risk of drug abuse.	[135–143]
Memantine	• noncompetitive NMDAR (weak voltage-dependent activity with fast-off kinetics)	• Inhibition of glycine receptors • 5-HT_3A_ antagonism	Generally well tolerated with no or minimal side effects Beware of the possibility of lowering the seizure threshold	[8, 144, 145]
Minocycline	• indirect inhibitory effect on excitotoxicity dependent on the NMDAR stimulation • modulation of the AMPAR and mGluR1 activity	• indirect inhibitory effect on excitotoxicity dependent on the stimulation of NMDA receptors • modulation of the activity of glutamatergic AMPA receptors and metabotropic mGluR1 receptors • Modulation of IL-1b activity • Inhibition of arachidonic 5-lipoxygenase • Inhibition of MMP-9 • Negative modulation of caspases	Generally well tolerated with side effect rate comparable to placebo The most frequent adverse events in minocycline groups were: gastrointestinal symptoms, flu-like symptoms, headache, and dizziness	[40–44, 146, 147]
Amantadine	• NMDAR antagonism	• D_2_ and D_1_ receptor agonism • antagonism towards neuronal receptor for acetylcholine • enhancing the release of dopamine and inhibiting its reuptake	No adverse effects were reported in depressive disorders add-on studies so far. Beware of the potential risk of QT prolongation and lowering the seizure threshold	[83–85, 148, 149]
Zinc	• NMDAR antagonism and modulation • mGluR1 and mGluR2 antagonism • ↑ AMPAR activity	• inhibition of GSK-3 • influence on the permeability of the blood-brain barrier. • influence on the activity of GPR39.	No adverse effects were reported in depressive disorder trials Long-term use of zinc salts may result in copper deficiency and anemia. Zinc is contraindicated in renal failure.	[58, 63, 65, 150–153]
*Crocus sativus* extract	• NMDAR antagonism	• sigma receptors antagonism • serotonin and noradrenaline reuptake inhibition • Interaction with GABA-A receptors? • Anti-inflammatory, anti-apoptotic, and oxidative stress-reducing effects. • MAO inhibition	Generally well tolerated The most common adverse effects reported in clinical trials for saffron supplementation were: headache, nausea, anxiety, and decreased appetite	[66, 68, 69, 78]
N-acetylcysteine	• Cystine/glutamate transporter activator • Glutamate NMDA receptor subunits activator	• Inhibitor of nuclear factor kappa-B kinase - Modulation of inflammatory / antiinflammatory processes	Generally well tolerated The most common adverse effects reported in clinical trials in depression or BD: altered energy levels, headaches, increased joint pain, heartburn, sweating, gastrointestinal symptoms the drug requires caution or should not be used in people with active peptic ulcer disease, severe respiratory failure or asthma, and esophageal varices	[100, 154–157]
Rapastinel	• Agonist of NMDAR glycine site		Generally well tolerated Transient dizziness was reported in one trial	[96, 98]
Basimglurant	• Nehative allosteric modulator of mGluR5		Generally well tolerated The most common adverse events in clinical trials in MDD were: dizziness, somnolence, headache	[110]

*GABA* - gamma-aminobutyric acid, *PD-L1* - programmed cell death protein ligand 1, *CoA* - coenzyme A, *COX* - cyclooxygenase, *CNS* - central nervous system, *MIPS* - myo-inositol monophosphate, *AMPAR* - alpha-amino-3-hydroxy-5-methyl-4-isoxazolepropionic acid, *NMDAR* - N-methyl-D-aspartic acid, *HCN* - hyperpolarization-activated cyclic nucleotide-gated channel, *cPLA2* - cytosolic phospholipase A2, *AA* - arachidonic acid, mGluR - metabotropic glutamate receptor, *D* - dopamine, *MAO* - monoamine oxidase

#### NMDA glycine site partial agonists

Rapastinel is a novel glutamatergic drug that acts as an agonist of the NMDAR glycine site [96]. Preclinical studies have shown that a single dose of rapastinel evoked antidepressant-like and procognitive effects that lasted for one week [97]. This activity has been linked with the NMDAR agonism and induction of NMDAR-dependent plasticity akin to long-term potentiation [96]. In a single double-blind, randomized, placebo-controlled study in a group of 116 TRD patients, a single injection of rapastinel significantly reduced depressive symptoms within two hours with an effect that lasted for seven days [98].

Apimostinel is a novel, selective modulator of NMDAR that presents several thousand-fold more potent than rapastinel at the glycine site [99]. Currently, the efficacy of intravenous and oral formulations of this drug is evaluated in phase II trial for MDD and phase I trial in healthy individuals, respectively [99].

### N-acetylcysteine

N-acetylcysteine (NAC) is commonly used as an antioxidant precursor to glutathione (γ-glutamylcysteinylglycine; GSH) which is used in the treatment of a wide range of medical conditions such as chronic obstructive pulmonary disease, paracetamol overdose and contrast-induced nephropathy [100]. A growing number of studies have pointed out that NAC may have a beneficial effect on several pathophysiological processes observed in major psychiatric disorders, such as neuroinflammation, disrupted glutamate neuronal activity, altered neurogenesis, oxidative stress, and increased apoptosis [101–103]. Clinical studies point out that NAC may improve psychopharmacotherapy outcomes in SZ [104]. Meta-analysis of 11 double-blind randomized placebo-controlled studies presented that this drug outperformed the placebo in ameliorating positive and negative symptoms, as well as deficits of attention and working memory in SZ patient groups [104]. In the case of affective disorders results of clinical trials are less encouraging. Systematic reviews and meta-analyses of double-blind randomized placebo-controlled trials pointed out that adjunctive treatment with the use of NAC has no beneficial effects in bipolar depression [105, 106], MDD [106] as well as on depressive symptoms in SZ patients groups [104].

### Basimglurant

Basimglurant is a novel, selective, negative allosteric modulator of mGluR5 that does not demonstrate pharmacological activity on monoamine reuptake transporters [107]. It is hypothesized that this drug leads to the preferential inhibition of mGluR5 in the subpopulation of cortical limbic GABA interneurons resulting in the disinhibition of glutamatergic networks in brain circuits related to mood regulation [108]. Preclinical studies indicated that basimglurant reduces the severity of depressive-like symptoms measured in forced swim test procedures and normalizes anhedonia index in animal models of anhedonia [107]. In a six-week-long double-blind placebo-controlled randomized clinical trial of 333 MDD patients, basimglurant did not differ in terms of the primary endpoint (mean change in clinician-rated Montgomery- Åsberg Depression Rating Scale (MADRS) score) [109]. However secondary and exploratory endpoint analyses demonstrated that this drug outperformed the placebo in the case of patient-rated measures of depressive symptoms (Quick Inventory of Depressive Symptomatology Self-Report and patient-rated MADRS) [109].

## Conclusions

The observation of rapid, robust, and long-lasting effects of NMDAR antagonists such as ketamine and esketamine encourages further research on drugs targeting glutamatergic transmission [5, 8]. A growing number of studies support the use of memantine and minocycline in MDD and SZ [8, 53, 54]. Amantadine, zinc, and *Crocus sativus* extracts yield the potential to ameliorate depressive symptoms in patients with affective disorders [60, 72, 84, 85]. Drugs with mechanisms of action based on glutamate constitute a promising pharmacological group in the treatment of mental disorders that do not respond to standard methods of therapy [1]. Further research is needed on their efficacy, safety, dosage, interactions, and side effects, to determine their optimal clinical use. It is also important to highlight that unconventional drugs like minocycline, zinc, or *Crocus sativus* extracts exhibit a broad spectrum of mechanisms of action [40, 41, 45, 56, 60, 66, 71]. Although these substances interact with glutamate receptors, it is currently unclear to what extent the clinical effects of these drugs are dependent on their effects on the glutamatergic system. Future research should consider the potential of modulating glutamatergic transmission through interactions not only with glutamate receptors but also with its transporters. Dysfunctions of excitatory amino acid transporters (EAATs) are involved in the pathophysiology of various neuropsychiatric disorders such as SZ, Alzheimer’s disease, autism spectrum disorder, or epilepsy [156–158]. EAAT2, the primary transporter responsible for glutamate clearance in the brain, is crucial for regulating glutamatergic transmission and preventing excitotoxicity [156]. There are ongoing studies that aim to identify novel positive allosteric modulators of EAAT2, which could offer innovative approaches for the development of therapies based on the enhancement of glutamate transport [156, 159]. Our review points out the crucial need for further research in the area of glutamatergic-based psychotropic drugs which could provide effective and safe alternatives.

## Data Availability

No datasets were generated or analysed during the current study.

## Abbreviations

AA: Arachidonic acid
AMPAR: Alpha-amino-3-hydroxy-5-methyl-4-isoxazolepropionic acid receptor
BD: Bipolar disorder
BDNF: Brain-derived neurotrophic factor
CANMAT: Canadian Network for Mood and Anxiety Treatments
cPLA2: Cytosolic phospholipase A2
CNS: Central nervous system
CoA: Coenzyme A
COX: Cyclooxygenase
D: Dopamine
DAT: Dopamine transporter
GABA: Gamma-aminobutyric acid
HCN: Hyperpolarization-activated cyclic nucleotide-gated channel
iGluR: Ionotropic glutamate receptor
MAO: Monoamine oxidase
MDD: Major depressive disorder
MIPS: Myo-inositol phosphate
mGluR: Metabotropic glutamate receptor
NMDAR: N-methyl-D-aspartate receptor
OCD: Obsessive-compulsive disorder
PCP: Phencyclidine
PD-L1: Programmed cell death protein 1 ligand
SZ: Schizophrenia
TRD: Treatment-resistant depression
MADRS: Montgomery-Åsberg Depression Rating Scale
EAAT: 2-Excitatory Amino Acid Transporter 2
